# Facilitated Synthesis of Mg_2_Ni Based Composites with Attractive Hydrogen Sorption Properties

**DOI:** 10.3390/ma14081936

**Published:** 2021-04-13

**Authors:** Eli Grigorova, Petar Tzvetkov, Stanislava Todorova, Pavel Markov, Tony Spassov

**Affiliations:** 1 Institute of General and Inorganic Chemistry, Bulgarian Academy of Sciences, Bl.11, Acad. G. Bonchev Str., 1113 Sofia, Bulgaria; egeorg@svr.igic.bas.bg (E.G.); tzvetkov@svr.igic.bas.bg (P.T.); pvlmarkov@svr.igic.bas.bg (P.M.); 2 Faculty of Chemistry and Pharmacy Sofia University “St. Kliment Ohridski”, 1 James Bourchier Blvd., 1164 Sofia, Bulgaria; nhtst@chem.uni-sofia.bg

**Keywords:** Mg_2_Ni based composite, complex hydride Mg_2_NiH_4_, hydrogen storage, Ni/MH battery

## Abstract

Composites based on Mg_2_Ni with 5% activated carbon from apricot stones (ACAP) have been prepared by ball milling and subsequent annealing in hydrogen atmosphere. The purpose of the primary metal (Mg, Ni, and V) milling was to reduce the particle size and achieve a good contact between them, without forming intermetallic compounds. During hydriding/dehydriding at 300 °C the amount of the Mg_2_Ni phase progressively increased, and after 10 cycles about 50% Mg_2_(Ni,V) was achieved. The hydrogenation produced mainly Mg_2_NiH_4_, but small amounts of MgH_2_ and VH_x_ were also detected in the powder mixture. Relatively high hydrogen storage capacity and fast hydriding/dehydriding kinetics of the Mg_2.1_Ni_0.7_V_0.3_—5 wt.% ACAP composite were determined both from hydrogen gas phase and electrochemically.

## 1. Introduction

Hydrogen is an energy carrier of the future, but its broad application depends on resolving some very important issues like transportation and storage. Storing hydrogen in form of metal or intermetallic hydrides provides a simple and safe solution. The Mg -based materials for hydrogen storage are characterized by high absorption capacity, good reversibility, abundance, and low price. The reduction of their thermodynamic stability is the subject of many investigations. The ternary hydride Mg_2_NiH_4_ of intermetallic Mg_2_Ni can be synthesized by ball milling and has sufficiently high absorption capacity as well as improved kinetics in comparison to Mg. Structural transformations causing color and property changes in Mg_2_NiH_4_ observed and described by some authors are of interest as well [1,2,3,4]. Extensive studies on the formation and decomposition of this hydride were also carried out by some other authors such as Li et al. [5,6,7], Cermak et al. [8,9,10], Orimo et al. [11,12], Varin et al. [13,14], and others [15,16,17,18,19,20,21]. Hydrogen absorption by Mg_2_Ni leads to the formation of several phases—two low-temperature with monoclinic and orthorhombic crystal structures and a high-temperature one with a cubic structure. The most popular methods of ternary hydride Mg_2_NiH_4_ type phase preparation are ball milling of Mg and Ni to obtain Mg_2_Ni and then hydrogenation [8,9,10] or ball milling of MgH_2_ and Ni in hydrogen [11,12,16,20] or combustion synthesis [5,6,7,15]. Mg_2_Ni is considered to be a promising hydrogen storage material for the future, which can be used as cathode in Ni-MH batteries. The theoretical electrochemical specific capacity of Mg_2_Ni as a negative electrode in Ni/MH batteries is reported to be about 1000 mAh/g. This value is almost threefold higher than this of MmNi_5_ and twofold higher than this of Zr(VNi)_2_. The poor hydriding kinetics at room temperature, easy oxidation of Mg_2_Ni, and low electrochemical cycle stability, however, seriously limit the possibilities of its application in Ni–MH batteries. Many efforts are concentrated on the improvement of the electrochemical characteristics of Mg_2_Ni based alloys [22,23,24,25,26,27,28,29].

Based on our experience of the hydrogen sorption and electrochemical hydriding of Mg_2_Ni type alloys [30,31,32,33,34,35], the present work is focused on the synthesis of the ternary hydride Mg_2_NiH_4_ at lower temperature and pressure than reported in previous studies [18,19,20]. The synthesis procedure includes ball milling of the metals and subsequent hydriding/dehydriding at 300 °C. The effects of different amounts of Mg excess and the addition of vanadium were revealed as well. Carbon containing additive (activated carbon prepared from waste agricultural by-product) was also applied to prevent the powder mixture from oxidation and agglomeration during ball milling. The hydrogen sorption properties of the as-prepared composites with overall compositions Mg_2.05_Ni_0.7_V_0.3_—5 wt.% ACAP (activated carbon derived from apricot stones) and Mg_2.1_Ni_0.7_V_0.3_—5 wt.% ACAP were characterized both from hydrogen gas phase and by electrochemical hydrogen charge/discharge.

## 2. Materials and Methods

Activated carbon was prepared by steam pyrolysis from apricot stones. The raw material was heated to carbonization temperature of 500 °C, in a stainless-steel vertical reactor placed in a tube furnace. After cooling down to ambient temperature, the solid product was activated with water vapor at 700 °C for 60 min. The activated carbon synthesis details can be found in [35]. The obtained activated carbon, Ni powder less than 150 μm with a 99.99% purity and V powder 325 mesh with a 99.5% purity purchased from Sigma Aldrich (Munich, Germany) were used for the preparation of the mixtures. Mg powders with purity 99% and 325 mesh or 50 mesh were purchased from Strem Chemicals (Newburyport, MA, USA). Mg_x_Ni_0.7_V_0.3_—5 wt.% ACAP, where x = 2.05 or 2.1 composites were synthesized by ball milling under Ar atmosphere, followed by annealing in hydrogen atmosphere. High purity argon (99.999%) and hydrogen (99.99%) purchased from Messer (Sofia, Bulgaria) were used for the experiments. Mixtures with composition Mg_2.05_Ni_0.7_V_0.3_ with 325 mesh Mg and Mg_2.1_Ni_0.7_V_0.3_ with 50 mesh Mg were prepared and 5 wt.% activated carbon (ACAP) was added subsequently. Both mixtures were ball milled under argon in a planetary mono mill Pulverisette 6 Fritsch Weimar (Thuringia, Germany) using the following conditions: ball to sample weight ratio 10:1, stainless steel balls with diameter 10 mm and weight 4 g; five balls in total were used for 2 g of sample mass, vial volume around 80 cm^3^, rotation speed of 200 rpm, and duration 180 min.

Hydrogen absorption-desorption characteristics were studied at various temperatures using self-constructed Sievert type apparatus. A detailed description of the volumetric method for hydrogen sorption measurements is given in [36]. After milling and hydrogenation, the samples were characterized by Transmission electron microscopy high resolution scanning transmission electron microscopy JEOL JEM 2100 (Akishima, Tokyo, Japan) with GATAN Orius 832 SC1000 camera that contains a charged-coupled device (Pleasanton, CA, USA) and X-ray diffraction phase analysis using Powder X-ray Diffractometer Bruker D8 Advance with a LynxEye detector and with Cu Kα radiation, vertical θ/θ goniometer, and a step size of 0.02 (2θ).

The electrochemical hydrogen charge/discharge behavior of the composites was studied using a three-electrode cell, allowing precise control of the electrodes’ geometry. The working electrode was prepared using 100 mg of the synthesized materials, 70 mg teflonized carbon, and 0.5 mL heptane. The mixture was pressed at about 130–150 atm to form a stable electrode, which was then dried in air. NiOOH/Ni(OH)_2_ was used as a counter electrode and Ag/AgCl as a reference. Each electrode was charged for 2 h at 20 mA and discharged to 450 mV at 1 mA in a 6 mol/dm^3^ KOH water solution.

## 3. Results and Discussion

**Figure 1:**
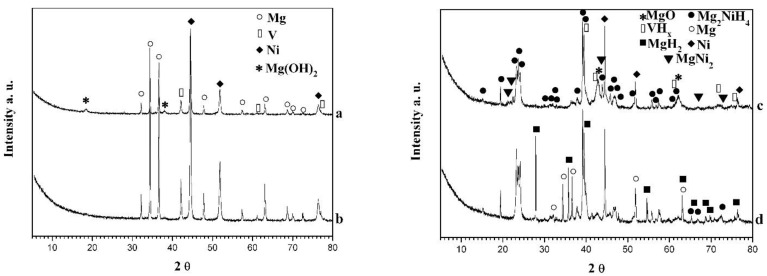
X-ray diffraction patterns of: (**a**) Mg_2.05_Ni_0.7_V_0.3_—5 wt.% ACAP ball milled; (**b**) Mg_2.1_Ni_0.7_V_0.3_—5 wt.% ACAP ball milled; (**c**) Mg_2.05_Ni_0.7_V_0.3_—5 wt.% ACAP hydrided at 300 °C and 1MPa and (**d**) Mg_2.1_Ni_0.7_V_0.3_—5 wt.% ACAP hydrided (curves (**c**,**d**) are obtained after 10 hydriding/dehydriding cycles and next hydriding).

X-ray diffraction (XRD) analysis of ball milled and hydrided samples for 1 h at 300 °C and 1 MPa are presented in Figure 1. The presence of the initial metals Mg, Ni, and V is detected in both samples after ball milling, without a clear formation of intermetallic phases. The diffraction peaks of the metals show some broadening due to the microstructural (crystallites) refinement and possible defects (dislocations and stacking faults) caused by the milling treatment. Subsequent annealing under argon of the powder mixtures at 300 °C for 2 h did not result in Mg_2_Ni formation as well. Only after 10 hydriding/dehydriding cycles at 300 °C the monoclinic and orthorhombic Mg_2_NiH_4_ ternary hydrides are detected, together with some unreacted Mg and Ni. Hayakawa et al. [4] obtained stabilization of the second low-temperature orthorhombic phase of ternary hydride Mg_2_NiH_4_ by substitution of Ni with 10 wt.% Co. The hydrided composites also contain some MgH_2_ and vanadium hydride phases. Moreover, some of the hydride phases (mainly Mg_2_NiH_4_) reveal nanocrystalline microstructure. MgH_2_ is clearly more visible in the composite with higher excess of magnesium. It is known that it is challenging to synthesize pure Mg_2_Ni or Mg_2_NiH_4_, and very often excess Ni, Mg, MgH_2_, as well as MgNi_2_ are detected [18,19,20,31,32,33,34].

By using DiffracPlus EVA program [37] and RIR (reference intensity ratio) method , a semiquantitative phase analysis for the composite Mg_2.1_Ni_0.7_V_0.3_—5 wt.% ACAP was made, showing: MgH_2_—11%, Mg_2_NiH_4_—45%, VH_x_—1–2%. Unreacted Ni and some Mg were also detected in the powder mixture. Practically, MgH_2_ was not detected in the hydridied composite Mg_2.05_Ni_0.7_V_0.3_—5 wt.% ACAP and the amount of the ternary hydride is slightly lower compared to that of the Mg richer material. The presence of vanadium hydride phase in the composites after cycling in hydrogen atmosphere means that such phase contributes to the overall hydrogen capacity and hydrogenation behavior. The PCT (pressure-composition-temperature) diagram of vanadium has two plateaus—one with lower hydrogen content corresponding to VH and a second one corresponding to VH_2_. The hydrogen storage capacity of vanadium is around 4 wt.%, but during cycling it drops to 2 wt.% due to the higher stability of hydride with lower hydrogen content. Numerous studies are dedicated to the hydriding of vanadium [31,38,39] and it is found that nonstohiometric hydride of vanadium VH_x_ plays the role of hydrogen pump and in this way facilitates the dissociative hydrogen chemisorption [31,39].

**Figure 2:**
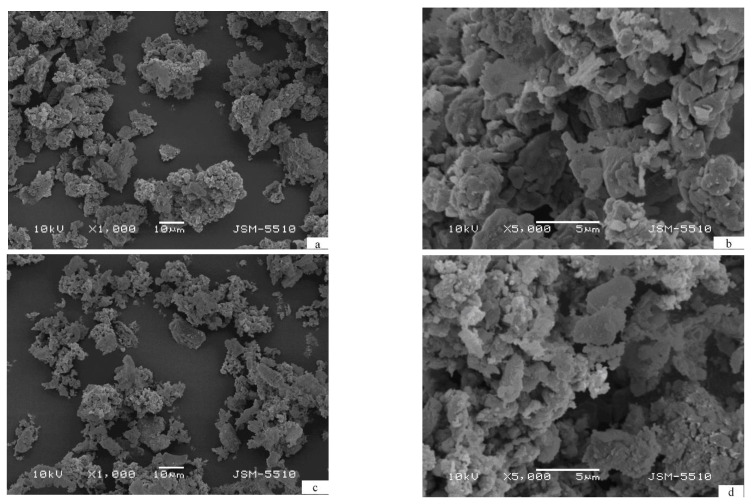
Scanning electron microscopy (SEM) micrographs of the composites after 10 hydriding/dehydriding cycles—(**a**,**b**) Mg_2.1_Ni_0.7_V_0.3_—5 wt.% ACAP and (**c**,**d**) Mg_2.05_Ni_0.7_V_0.3_—5 wt.% ACAP.

The morphology of the cycled two composites does not differ significantly. As a result of ball milling and next hydriding/dehydriding the particles of both composites reveal similar shape and size, which can be averaged as 10 μm (Figure 2). The larger particles contain dome cracks, a result of powder pulverization during hydriding/dehydriding (Figure 2b,d).

**Figure 3:**
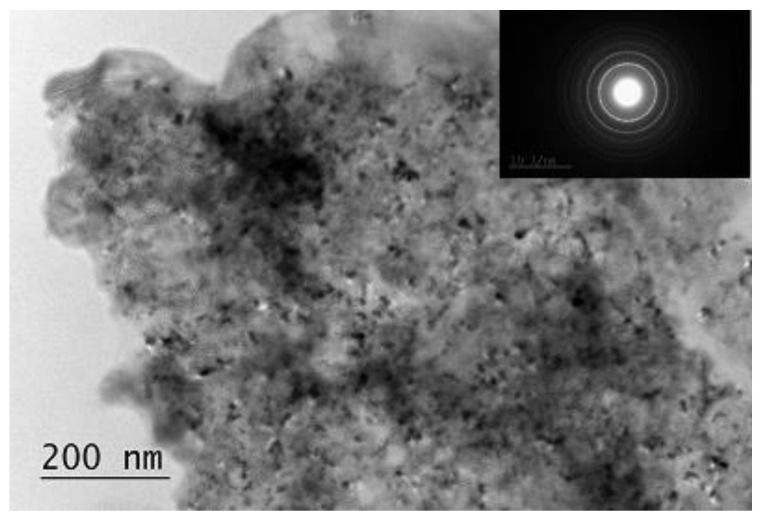
Transmission electron microscopy (TEM) image and bright field micrograph SAED pattern of the Mg_2.05_Ni_0.7_V_0.3_—5 wt.% ACAP after hydriding.

The transmission electron microscopy (TEM) analysis of the composites is in good agreement with the X-ray diffraction (XRD) patterns. The composites consist of nanocrystallites and the polycrystalline SAED (Selected area electron diffraction) shows the presence of mainly Mg_2_NiH_4_ phase with monoclinic and orthorhombic structures (Figure 3). Additionally, some graphite, MgH_2_, Ni, VH_x_ are also detected.

**Figure 4:**
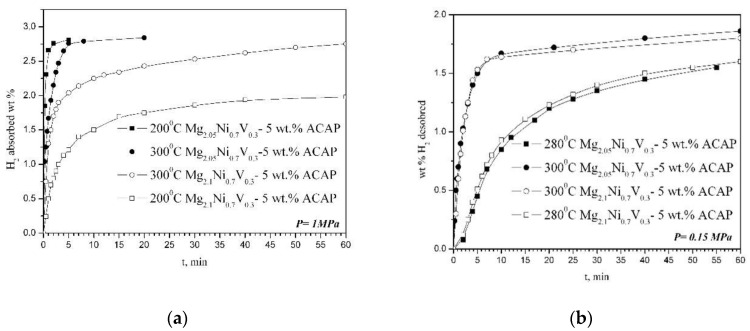
Hydrogen (**a**) absorption and (**b**) desorption kinetic curves.

The hydrogen sorption characteristics of the two composites are studied at 200 and 300 °C and a pressure of 1 MPa during absorption, and 300 and 280 °C and a pressure of 0.15 MPa for desorption (Figure 4). For both samples, 10 cycles of absorption and desorption are performed, and the best results are presented in Figure 4. The hydrogen absorption capacity of Mg_2.05_Ni_0.7_V_0.3_—5 wt.% ACAP obtained after only 5–10 min at 300 °C is around 2.8 wt.% H_2_. The achieved final absorption capacity after 60 min of hydriding for both samples at 300 °C is practically the same. The Mg richer composite contains more MgH_2_, which is clearly seen on the XRD patterns (Figure 1). It is known that Mg hydriding kinetics is slower than that of Mg_2_Ni. Furthermore, this composite has also a little higher Mg_2_NiH_4_ content, characterized with better hydriding kinetics, but lower hydrogen capacity. Additionally, in the Mg_2.05_Ni_0.7_V_0.3_—5 wt.% ACAP composite some amount of MgNi_2_ is also detected, which is known to be favorable for promoting the diffusion of hydrogen and thus enhances the hydrogen sorption kinetics of MgH_2_. All above mentioned factors could explain the observed difference in the kinetic curves (Figure 4). The desorption curves at 300 and 280 °C are very similar for the two compositions and practically overlap. For 60 min the composites desorb more than 50% of the absorbed hydrogen. The hydrogen desorption rate at 280 °C is lower for both composites. Apparently, the very fast hydrogen absorption at 300 °C reaching about 3 wt.% H_2_ for Mg_2.1_Ni_0.7_V_0.3_—5 wt.% ACAP can be considered as a promising result that can be upgraded by optimizing the phase composition and microstructure of the composite.

**Figure 5:**
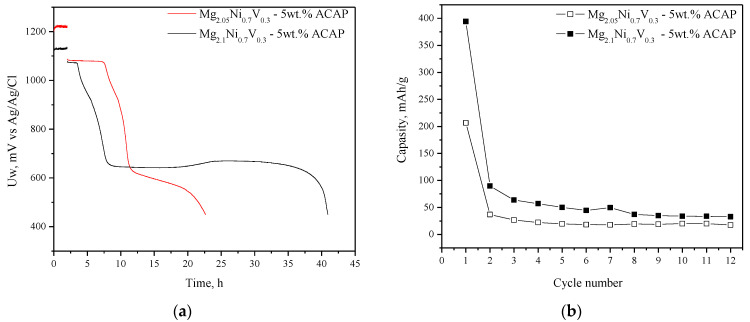
Discharge capacity vs. cycle number (**a**) and first charge and discharge curves (**b**).

The already activated composites (after 10 absorption/desorption cycles at 300 °C and 1 MPa/0.15 MPa H_2_, respectively) were tested as negative electrodes in a Ni/MH battery. Figure 5 compares the electrochemical hydrogen charge/discharge behavior of the two composites at galvanostatic conditions. A relatively high initial discharge capacity of 400 mAh/g was obtained for the Mg richer composite (Mg_2.1_Ni_0.7_V_0.3_—5 wt.% ACAP) and about 200 mAh/g for Mg_2.05_Ni_0.7_V_0.3_—5 wt.% ACAP. Obviously, the higher capacity of the Mg richer composite has to be associated with the larger amount of the Mg_2_Ni phase formed under the applied synthetic conditions (proved by XRD, Figure 1). The achieved discharge capacity of 400 mAh/g is relatively high in comparison to others mentioned in literature concerning similar materials [24,26,28]. The specific discharge capacity of about 470 mAh/g was achieved by nanocrystalline alloy Mg_1.95_Y_0.05_Ni_0.92_Al_0.08_ as negative electrode in Ni/MH battery [24]. S. Pedneault et al. reported that nanostructured Mg_2_Ni materials prepared by cold rolling and used as negative electrode for Ni–MH batteries shows initial discharge capacity of 205 mAh/g [26]. As can be seen in Figure 5, very rapid degradation of both materials is observed with a capacity decay of about 90% of the initial discharge capacity. The rapid decrease of electrochemical capacity with cycling is well known for Mg-based materials and it is attributed to Mg(OH)_2_ formation. The layer of Mg(OH)_2_ on the electrode limits the hydrogen absorption/desorption reaction and in addition is consuming the active material. For example, in the paper published by Pedneault et al., after only five cycles the discharge capacity is reduced from 231 to ∼40 mAh/g [26]. Some further studies and attempts to improve the stability of the electrochemical capacity of the studied composites, including appropriate corrosion resistant coatings, are underway.

## 4. Conclusions

Simple and easy synthesis procedure of the ternary hydride Mg_2_NiH_4_ is presented, including ball milling of the metal powders (Mg, Ni, and V), followed by gas phase hydriding/dehydriding at 300 °C. To prevent the metal powders from oxidation and agglomeration during milling activated carbon is also added. Thus, two composites with different excess of Mg are prepared: Mg_2.05_Ni_0.7_V_0.3_—5 wt.% ACAP and Mg_2.1_Ni_0.7_V_0.3_—5 wt.% ACAP. For the composite containing higher excess of Mg (Mg_2.1_Ni_0.7_V_0.3_—5 wt.% ACAP), larger amount of Mg_2_Ni phase is detected.

Fast hydrogen absorption and desorption kinetics are obtained at 300 °C, keeping relatively high hydrogen capacity of 2.8 wt.% H_2_. The desorption rate and the capacity are very close for the two composites at 300 °C and 280 °C. Higher electrochemical discharge capacity of 400 mAh/g is measured for Mg_2.1_Ni_0.7_V_0.3_—5 wt.% ACAP, which later decreases due to oxidation of the active electrode material. Subsequent attempts to improve the corrosion resistance of the studied composites, including appropriate corrosion resistant coatings, are underway.

## Data Availability

Data is contained within the article.
